# ECG Abnormalities and Arterial Stiffness by HIV Status among High-Risk Populations in Rakai, Uganda: A Pilot Study

**DOI:** 10.5334/gh.1015

**Published:** 2021-12-08

**Authors:** Rocio Enriquez, Robert Ssekubugu, Godfrey Kigozi, Dorean Nabukalu, Gaetano Marrone, Susanne Rautiainen, Bruna Gigante, Steven J. Reynolds, Fred Nalugoda, Larry W. Chang, Anna Mia Ekström, Nelson K. Sewankambo, David Serwadda, Helena Nordenstedt

**Affiliations:** 1 Department of Global Public Health, Karolinska Institutet, Stockholm, SE; 2 Rakai Health Sciences Program, Kalisizo, UG; 3 Venhälsan, Södersjukhuset, Stockholm, SE; 4 Department of Medicine, Karolinska Institutet, Stockholm, SE; 5 Laboratory of Immunoregulation, Division of Intramural Research, National Institute for Allergy and Infectious Diseases, National Institutes of Health, Bethesda, Maryland, US; 6 Department of Epidemiology, Johns Hopkins Bloomberg School of Public Health, Baltimore, Maryland, US; 7 Division of Infectious Diseases, Department of Medicine, Johns Hopkins School of Medicine, Baltimore, Maryland, US; 8 Department of Medicine, Makerere University School of Medicine, Kampala, UG; 9 Department of Disease Control and Environmental Health, Makerere University School of Public Health, Kampala, UG; 10 Division of Internal Medicine, Danderyd University Hospital, Karolinska Institute, Stockholm, SE

**Keywords:** Cardiovascular disease, HIV, ECG, Arterial Stiffness, Left Ventricular Hypertrophy, and Uganda

## Abstract

**Background:**

People living with HIV are at increased risk for cardiovascular disease (CVD). In sub-Saharan Africa, population-based data on major CVD events such as stroke and myocardial infarction are difficult to collect. The use of proxy measures could be a feasible way to better study CVD in such settings. This study aimed to determine the acceptance of incorporating ECG and arterial function measurements into a population-based cohort study and to assess the prevalence of ECG abnormalities and arterial stiffness.

**Methods:**

A pilot study was conducted within the Rakai Community Cohort Study in Uganda on two high-risk CVD populations; one determined by age (35–49) and Framingham CVD risk scores and the other by age alone (50+). Data on ECG, arterial function, blood pressure, and HIV status were collected. The acceptability of incorporating ECG and arterial function measurements was established as an acceptance rate difference of no more than 5% to blood pressure measurements.

**Results:**

A total of 118 participants were enrolled, 57 participants living with HIV and 61 HIV-negative participants. Both ECG measurements and arterial function were well accepted (2% difference). Left ventricular hypertrophy (LVH) and arterial stiffness (>10 m/s) were common in both participants living with HIV and HIV-negative participants across the two high-risk populations. Prevalence rates ranged from 30% to 53% for LVH and 25% to 58% for arterial stiffness. Arterial stiffness at the 11 m/s cutoff (p = 0.03) was found to be more common among participants living with HIV in the 35–49 population.

**Conclusions:**

The incorporation of ECG and arterial function measurements into routine activities of a population-based cohort was acceptable and incorporating these proxy measures into cohort studies should be explored further. LVH and arterial stiffness were both common irrespective of HIV status with arterial stiffness potentially more common among people living with HIV.

## Introduction

With the global expansion of antiretroviral therapy (ART) in the past decades, HIV has turned from a fatal disease to a manageable chronic disease. As people living with HIV live longer, HIV care services will need to be adapted to address age-related diseases [1,2]. Previous studies have demonstrated that people living with HIV have a 1.5–2 fold increased risk for cardiovascular disease (CVD) as compared to the general population [3,4,5,6,7,8]. Evidence is, however, primarily derived from North America and Europe, high-income settings, while 70% of the global HIV population lives in low-and middle income settings in sub-Saharan Africa [9]. Given the context and population-specific variations that exist in sub-Saharan Africa with regard to genetics, diet, physical activity, and other chronic infections coupled with low access and delivery of health services targeting CVD, it is critical to understand the prevalence and implications of CVD among people living with HIV in these settings.

Many countries across sub-Saharan Africa have fragmented health-information systems that either generate partial or no data in relation to CVD in both the general population and people living with HIV [10]. Large-scale population-based cohorts can, in this environment, be used to provide valuable information on disease burden, etiology, and viable prevention and treatment interventions to subsequently inform health policy and service delivery. The lack of data for key CVD outcomes of stroke and myocardial infarction (MI), likely due to the inherent difficulties in measuring these outcomes in settings where most of these events go undetected by the health-care system [11], is an area in which subclinical predictors could potentially serve as a proxy. The 12-lead electrocardiogram (ECG) and non-invasive tools for arterial function are possible measurements for CVD prediction that can potentially be incorporated in population-based cohort studies.

The ECG is a widely used clinical tool in the evaluation of CVD while non-invasive tools to measure arterial function, such as pulse wave arteriography, are increasingly being used in research practice and clinically. Both tools are relatively inexpensive, portable, and easy to use, making them ideal for use in resource-poor settings and large cohort studies. Finally, both ECG abnormalities and arterial stiffness, the primary measurement for arterial function, have been found to be independent predictors of CVD outcomes (stroke and MI) and mortality [12,13].

We conducted a pilot study within the Rakai Community Cohort Study (RCCS) in south-central Uganda to investigate the acceptability of obtaining ECG and arterial function measurements from participants in a large, population-based cohort study. We also explored the prevalence of key ECG and arterial stiffness measurements by HIV status among two high-risk populations, one determined by age and Framingham CVD risk score and the other by age alone.

## Methods

### Participant Selection and Recruitment

The pilot study was implemented from October 2018 to January 2019 across five communities of the RCCS. The design and methods of the RCCS have been extensively described elsewhere [14,15]. In summary, the RCCS is an open population-based cohort study in 40 communities in Rakai, Uganda, that implements 1) annual census on all community inhabitants regardless of age and 2) survey rounds, including HIV testing, on all consenting participants aged 15–49 years old. The communities for the pilot study were conveniently selected to align to routine data collection of the 19^th^ survey round of the RCCS.

**Figure 1:**
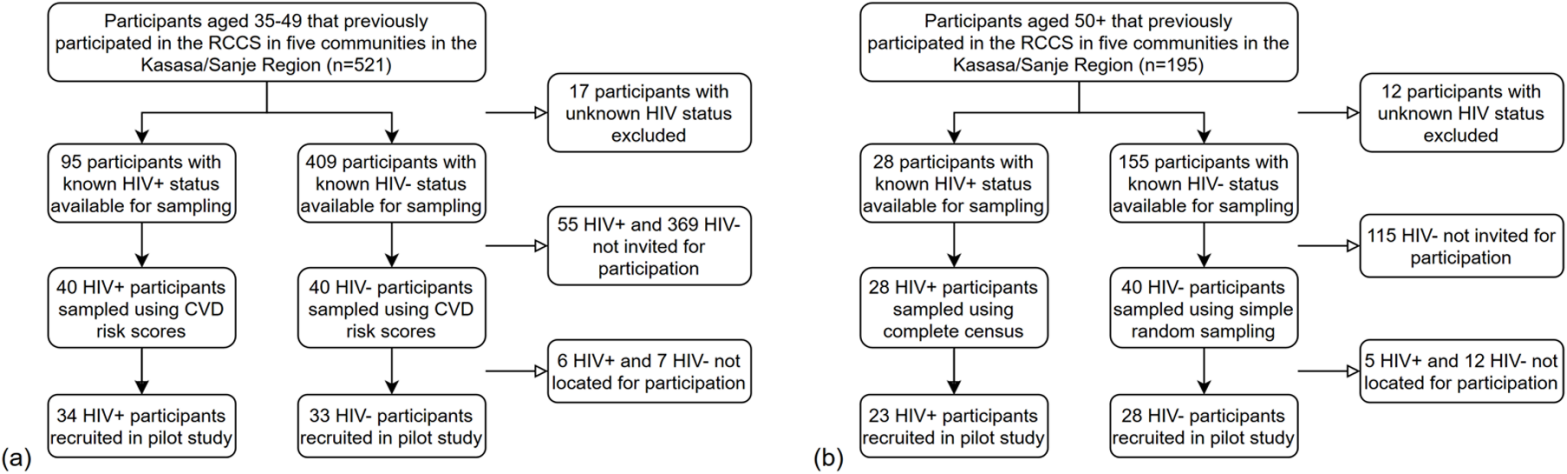
Participant Selection and Recruitment by High-Risk Population of Interest: A) 35–49 Population B) 50+ Population.

The sample populations for the pilot study were selected using two different recruitment strategies for high-risk populations, one based on age and CVD risk stratification while the other based on age alone. These recruitment strategies were selected as CVD risk and increasing age are predictive of CVD events and in considerations of the cohort’s characteristics [16,17]. The first high-risk population consisted of individuals of the five targeted communities from the oldest age group (35–49 years of age) of the RCCS, that had participated in the previous survey round (18^th^ survey round) with a known HIV status (94 people living with HIV and 409 HIV-negative). Non-laboratory-based Framingham Ten-Year General Cardiovascular Disease Risk Scores were then calculated for each individual [18]. Information used to calculate the scores was collected in the previous survey round and included sex, age, systolic blood pressure, current treatment for hypertension, current smoking status, diabetes status, and body mass index (BMI). Individuals were then stratified by HIV status, with the top 40 individuals with the highest CVD risk scores selected for possible participation. The second high-risk population consisted of individuals of the five targeted communities aged 50+ years as identified from the census round that proceeded the 19^th^ survey round of the RCCS. For possible inclusion, participants had to have previously participated in the RCCS before turning 50 years with a known HIV status (28 people living with HIV and 155 HIV-negative). Given the limited number of individuals living with HIV in the 50+ population, a complete census was used to select all 28 individuals while simple random sample was used to select 40 individuals without HIV for possible participation. The two sampling populations totaled 148 possible participants for recruitment: 80 individuals in the two 35–49 population sub-groups and 68 individuals in two 50+ population sub-groups. Figure 1 details participant selection and recruitment further.

### Data Collection

ECG and arterial function measurements were captured in parallel with routine RCCS data collection procedures. Selected participants were invited to a separate and private tent for the capture of both measurements after completion of the blood pressure measurements which was taken at another station within the RCCS field data collection operations. ECG measurements were first captured among selected participants using a SE-12000 Express ECG machine (Edan Instruments, Shenzhen, China). All measurements were taken in the supine position with electrodes placed according to the standard 12-lead ECG locations. A repeated ECG was performed if a participant moved during the measurement, an electrode detached, or if the ECG report stated that the ECG was abnormal. For the participant, no more than five minutes were needed to capture the measurement. Approximately three to five minutes after the completion of the ECG, arterial function measurements were captured among selected participants. The Arteriograph (TensioMed Ltd, Budapest, Hungary) was used to capture the aortic pulse wave velocity, a measurement shown to be highly correlated at predicting coronary artery disease as compared to carotid-femoral pulse wave velocity, the current research standard as established by the European Network for Non-invasive Investigation of Large Arteries [19,20]. For this measurement, the distance between the jugulum and symphysis was captured in the supine position and measured at the end of the ECG measurement as the participant is in the supine position for its measurement. Arm circumference was then captured in the seated position right before the start of arterial function measurements. The distance between the jugulum and symphysis was needed by the software to calculate the pulse wave velocity, and arm circumference was needed to determine the appropriate arm cuff for the measurement. For the participant, between one to two minutes were needed to capture the measurement and felt similar to providing a blood pressure measurement. Up to three attempts were made to capture the arterial function measurement after waiting five minutes in-between measurements and with the participant seated with their legs uncrossed. On average an approximate ten to twelve minutes were needed to capture both measurements from entering to leaving the tent. In some instances, where challenges were experienced and particularly in capturing the arterial stiffness measurement, up to 25 minutes was needed to capture both measurements.

Participants also completed a sociodemographic and behavior interview based on a shortened version of the routine RCCS questionnaire. For blood pressure, two measurements were taken five minutes apart while the participant was seated with their legs uncrossed. Anthropometric measurements of weight, height, waist circumference, and hip circumference were taken while the participant was wearing light clothing.

For the interpretation of ECG reports, two independent reviewers (HN and SR) reviewed the reports based on a predefined protocol looking at ECG interval values and ECG abnormalities. For any discrepancy found between the classification of abnormalities a third reviewer (BG) acted as tiebreaker.

### Acceptability Threshold

The acceptability of incorporating ECG and arterial function measurements into the RCCS survey activities by cohort participants was defined prior to the start of the pilot as a difference of no more than 5% in the participant acceptance rate between ECG and arterial function measurements as compared to blood pressure measurements, which have been routinely collected within the RCCS for the last four data collection rounds.

### ECG and Arterial Function Definitions

The primary ECG abnormalities recorded were: signs of previous MI, QTc prolongation, left bundle branch block (LBBB), right bundle branch block (RBBB), first-degree atrioventricular (AV) block, second-degree AV block, third-degree AV block, and left ventricular hypertrophy (LVH). Previous MI was defined as a Q-wave in V2 or V3 >0.02 s or presence of a QS complex in V2 or V3 [21]. QTc prolongation was defined as a QT interval ≥0.47 s in women and ≥0.45 s in men. LBBB was defined as QRS complex ≥0.12 s plus a deep and wide S-wave in V1-V2, and a positive R-wave in V5-V6 (‘V-complex’). RBBB was defined as a QRS ≥0.12 s, a large R-wave in V1, and a wide and deep S-wave in V6 (‘M-complex’). First-degree AV block was defined as PQ interval ≥0.22 s or >five small boxes. Second-degree AV block was defined as 1:1 P-wave-to-QRS-complex ratio not maintained. Finally, a third-degree AV block was defined as AV dissociation, meaning that the P-waves occur at one rate and the QRS complexes at another. An abnormality of unknown origin was defined as an unknown abnormality. LVH was defined according to the Sokolow-Lyon and Total QRS criteria [22,23]. For Sokolow-Lyon, it was determined that LVH was present if the sum of the S-wave in V1 plus the R-wave in V5 or V6 ≥35 mm. For total QRS, LVH was determined to be present if the total sum of the QRS amplitude for each of the 12 leads was ≥175 mm.

For arterial function, the primary outcome of interest was arterial stiffness. Arterial stiffness was defined as a pulse wave velocity >9 m/s indicative of significant alteration in aortic function while poor peripheral arterial tone was defined as an augmentation index >33% [24]. Pulse wave velocity at >10 m/s and >11 m/s cutoffs were additionally explored.

### Other Definitions

Alcohol consumption, fruit and vegetable consumption, and current smoking status were all based on self-reported data obtained from the socio-demographic and behavior questionnaire. Alcohol consumption was dichotomized into frequent alcohol consumption, having consumed one or more alcoholic drinks within the past six days, and infrequent alcohol consumption, having consumed one or more alcoholic drinks seven or more days ago. Current smoking status was dichotomized as yes or no. Fruit and vegetable consumption was dichotomized into consuming below or the daily median consumption and above among the surveyed participants. Body Mass Index (BMI) was defined as <18.5 kg/m^2^ for underweight, ≥18.5–25 kg/m^2^ for normal, ≥25–30 kg/m^2^ for overweight, and ≥30 kg/m^2^ for obese [25]. Due to low numbers, the two categories for overweight and obese were combined to ≥25 kg/m^2^. Waist circumference was assessed according to Uganda national guidelines which are aligned to WHO guidelines [26,27]. At increased risk was defined as a waist circumference of 95–102 cm for males and 81–88 cm for females and substantially at increased risk was defined as ≥103 cm for males and ≥89 cm for females. A waist-to-hip ratio was also assessed using WHO guidelines with substantially at increased risk defined as ≥0.90 for males and ≥0.85 for females [25]. Hypertension was defined using Uganda national guidelines as the average of two blood pressure measurements with a systolic blood pressure ≥140 mmHg or a diastolic blood pressure ≥90 mmHg; or participant reporting being on hypertension medication [26].

### Statistical Analyses

Baseline characteristics and prevalence rates of ECG abnormalities and arterial stiffness measurements were tabulated by both high-risk population and HIV status. Differences in ECG and arterial function measurements by HIV status within each high-risk population were assessed by either two-tailed t-test, chi-square test, or fisher’s exact test. During the analysis phase, a secondary analysis was conducted to determine the potential impact of selecting study participants in the 35–49 population using the CVD risk score stratification method on ECG and arterial stiffness measurements. This was accomplished by comparing ECG and arterial stiffness prevalence rates within a sub-sample of ten participants with comparable CVD risk scores by HIV status. Stata 15 (Stata Corporation, College Station, USA) was used for all tabulations and analyses.

### Ethics

The pilot study was approved by the Research and Ethics Committee of the Uganda Virus Research Institute (GC/127/18/07/657), the Swedish Ethical Review Authority (2018/2542-31/2) and the Uganda National Council for Science and Technology (HS540). All participants provided written informed consent as part of the RCCS activities and were compensated for their time. For any ECG found to be ‘abnormal,’ a trained healthcare provider off-site reviewed the ECG and communicated both results and any next steps back to the study participant via the field staff. Field staff were trained on how to communicate the results of the Arteriograph in terms of lifestyle changes.

## Results

### Characteristics of Study Participants

Study participants characteristics by HIV status are reported in Table 1. A total of 118 participants were recruited into the pilot study. Among participants in the 35–49 population, 34 participants living with HIV and 33 HIV-negative participants were included. In this population, more males were recruited than females with the difference most pronounced among HIV-negative participants (88% males vs 12% females). Less frequent alcohol consumption (26% vs 58%) and higher percentage of currently smoking (94% vs 52%) were reported among participants living with HIV as compared to HIV-negative participants. Participants living with HIV were found to have a higher prevalence of being at substantially increased risk (27% vs 16%) for CVD from waist circumference measurements.

**Table 1:** Participant Characteristics by High-Risk Population and HIV Status.

	35–49 and HIV + No. (%)	35–49 and HIV- No. (%)	50+ and HIV + No. (%)	50+ and HIV- No. (%)

**Sex**	n = 34	n = 33	n = 23	n = 28
Female	14 (41%)	4 (12%)	11 (48%)	15 (54%)
Male	20 (59%)	29 (88%)	12 (52%)	13 (46%)
**Age**	n = 34	n = 33	n = 23	n = 28
Age (mean, SD in years)	46 (3.7)	47 (2.5)	56 (3.5)	60 (6.0)
**Education** ^1^	n = 32	n = 30	n = 22	n = 25
No education	3 (9%)	0 (0%)	1 (5%)	1 (4%)
Incomplete primary	6 (19%)	10 (33%)	6 (27%)	5 (20%)
Primary and above	23 (72%)	20 (67%)	15 (68%)	19 (76%)
**Alcohol Consumption**	n = 34	n = 33	n = 23	n = 28
Non-drinker	13 (38%)	12 (36%)	12 (52%)	11 (39%)
Infrequent drinker (last drink < 7 days)	12 (35%)	2 (6%)	9 (39%)	5 (18%)
Frequent drinker (last drink ≥ 7 days)	9 (26%)	19 (58%)	2 (9%)	12 (43%)
**Smoking Status**	n = 34	n = 33	n = 23	n = 28
Non-smoker	2 (6%)	16 (49%)	0 (0%)	3 (11%)
Current smoker	32 (94%)	17 (52%)	23 (100%)	25 (89%)
**Daily Fruit and Vegetable Consumption**	n = 34	n = 33	n = 23	n = 28
Consumption (servings, median)	1.2	1.5	1.4	1.4
Below average (<1.14 servings)	16 (47%)	14 (42%)	12 (52%)	12 (43%)
Above average (≥1.14 servings)	18 (53%)	19 (58%)	11 (48%)	16 (57%)
**BMI**	n = 34	n = 32	n = 23	n = 28
Underweight	5 (15%)	5 (16%)	3 (13%)	7 (25%)
Normal	21 (62%)	18 (56%)	13 (57%)	11 (39%)
Overweight and above	8 (24%)	9 (28%)	7 (30%)	10 (36%)
**Waist Circumference**	n = 34	n = 32	n = 23	n = 28
No increased risk	22 (65%)	21 (66%)	12 (52%)	15 (54%)
At increased risk	3 (9%)	6 (19%)	6 (26%)	4 (14%)
Substantially at increased risk	9 (27%)	5 (16%)	5 (22%)	9 (32%)
**Waist-to-hip Ratio** ^6^	n = 34	n = 32	n = 23	n = 28
No increased risk	21 (62%)	20 (62%)	12 (52%)	11 (39%)
Substantially at increased risk	13 (38%)	12 (38%)	11 (48%)	17 (61%)
**Blood Pressure Measurements**	n = 33	n = 32	n = 23	n = 28
Hypertension				
Normal (<120/80 mmHg)	13 (39%)	6 (19%)	6 (26%)	1 (4%)
Pre-hypertension (≥120/80 mmHg & (<140/90 mmHg)	11 (33%)	11 (34%)	6 (26%)	9 (32%)
Hypertension (≥140/90 mmHg or medication)	9 (27%)	15 (47%)	11 (48%)	18 (64%)
**Time Since HIV Diagnosis**	n = 34		n = 22	
Within last year	3 (9%)	–	5 (23%)	–
1–4 years	11 (32%)	–	7 (32%)	–
>4 years	20 (59%)	–	10 (46%)	–
**Currently on HIV Treatment**	n = 34		n = 22	
Yes	30 (88%)	–	12 (55%)	–

SD = standard deviation; BMI = body mass index; mmHg = millimetres of mercury. ^1^ Denominator excludes nine participants that responded yes to being educated but did not provide information on highest level of education.

Among participants in the 50+ population, 23 participants living with HIV and 28 HIV-negative participants were included. In this population, the median age among participants living with HIV was 56 years while for HIV-negative participants it was 60 years. Less frequent alcohol consumption (9% vs 43%) was reported among participants living with HIV as compared to HIV-negative participants. Participants living with HIV were found to have a BMI within the normal range as compared to HIV-negative participants (57% vs 39%) who had more underweight and overweight participants. Finally, hypertension was lower among participants living with HIV as compared to HIV-negative participants (48% vs 64%).

### Acceptability Threshold

The acceptance rates for arterial function and ECG measurements were 100% while the acceptance rate for blood pressure measurements was 98% resulting in a 2% (Confidence Interval 0.96–1.01) difference between the measurements across the two high-risk populations.

### ECG and Arterial Function Measurements

Both ECG and arterial function measurements captured are detailed in Table 2. In terms of ECG abnormalities, a total of 119 abnormalities were found in 69 participants across the two high-risk populations. LVH was the most common abnormality found with 52 identified by the Sokolow-Lyon criteria, and 53 identified by the QRS criteria. In the 50+ population it was more common to have an LVH identified by total QRS criteria among participants living with HIV as compared to among HIV-negative participants (43% and 30% respectively). No statistical difference was found by HIV status for any of the ECG abnormalities reviewed in either high-risk population.

**Table 2:** ECG and Arterial Function Measurements by High-Risk Population and HIV Status.

	35–49 and HIV + No. (%)	35–49 and HIV- No. (%)	P-value^4^	50+ and HIV + No. (%)^3^	50+ and HIV- No. (%)	P-value

**ECG Measurements**	n = 34	n = 33		n = 23	n = 28	
ECG interval values						
Heart rate (bpm, mean)	71.8	70.5	0.71	70.2	76.3	0.13
PR-interval (ms, mean)	159.0	157.8	0.82	110.8	111.6	0.02
QRS-interval (ms, mean)	86.4	89.3	0.27	87.0	89.0	0.57
QTc-interval (ms, mean)	426.4	418.2	0.18	424.0	438.7	0.01
ECG Abnormalities^1^						
Signs of Previous MI	0 (0%)	0 (0%)	–	1 (4%)	0 (0%)	0.45
QTc Prolongation	3 (9%)	2 (6%)	0.51	2 (9%)	3 (11%)	0.60
LBBB	0 (0%)	0 (0%)	–	0 (0%)	0 (0%)	–
RBBB	0 (0%)	0 (0%)	–	0 (0%)	0 (0%)	–
First-degree AV block	0 (0%)	0 (0%)	–	0 (0%)	2 (0%)	0.50
Second-degree AV block	0 (0%)	0 (0%)	–	0 (0%)	0 (0%)	–
Third-degree AV block	0 (0%)	0 (0%)	–	0 (0%)	0 (0%)	–
LVH by diagnostic criteria	n = 34	n = 33		n = 23	n = 27	
Sokolow-Lyon	17 (50%)	16 (49%)	0.90	9 (39%)	10 (37%)	0.88
Total QRS	18 (53%)	17 (52%)	0.91	10 (43%)	8 (30%)	0.31
**Arterial Function Measurement** ^s2^	n = 25	n = 20		n = 19	n = 10	
Augmentation index (mean)	25.9	30.4	0.37	30.4	36.5	0.36
Augmentation index (>33%)	7 (28%)	8 (40%)	0.40	9 (47%)	5 (50%)	0.89
Pulse wave velocity (m/s, mean)	10.2	9.4	0.12	10.0	9.9	0.79
Arterial stiffness (pulse wave velocity) prevalence^3^						
≥9 m/s	16 (64%)	13 (65%)	0.94	14 (74%)	8 (80%)	1
≥10 m/s	13 (52%)	5 (25%)	0.07	11 (58%)	4 (40%)	0.45
≥11 m/s	9 (36%)	1 (5%)	0.03	4 (21%)	1 (10%)	0.63

ECG = Electrocardiogram; Bpm = beats per minute; ms = milliseconds; MI = Myocardial infarction; LBBB = Left bundle branch block (LBBB); RBBB = Right bundle branch block (RBBB); AV block: atrioventricular block; LVH = Left ventricular hypertrophy; m/s = meters per second. ^1^ The table below does not show one AV block of unknown origin found in 50+ and HIV+ population. ^2^ Arterial function measurements admissible for use were captured in 74 participants (63%, out of 118 participants) of those that underwent the diagnostic measurement. ^3^ Lower prevalence cut-offs include participants categorized at higher cut-offs. ^4^ P-values derided from either t-test, pearsons chi-square, or fisher’s exact test using a null hypothesis that there is no difference between the two populations.

Arterial function measurements were captured in 74 out of the 118 participants (63%), while in the other 44 participants (37%) no reading was obtained despite several attempts. The prevalence of arterial stiffness was found to be greater among the participants living with HIV at increased arterial stiffness cutoffs among the 35–49 population. Among participants in this population and using the ≥10 m/s cutoff, 52% of participants living with HIV and 25% of HIV-negative participants were found to have arterial stiffness. At the ≥11 m/s cutoff, 36% participants living with HIV and 5% of HIV-negative participants were found to have arterial stiffness. A statistical difference was found by HIV status at the ≥11 m/s cutoff among the 35–49 population (p = 0.03) and no statistical difference was found in the 50+ population for any of the cutoffs reviewed.

### Secondary Analysis

**Figure 2:**
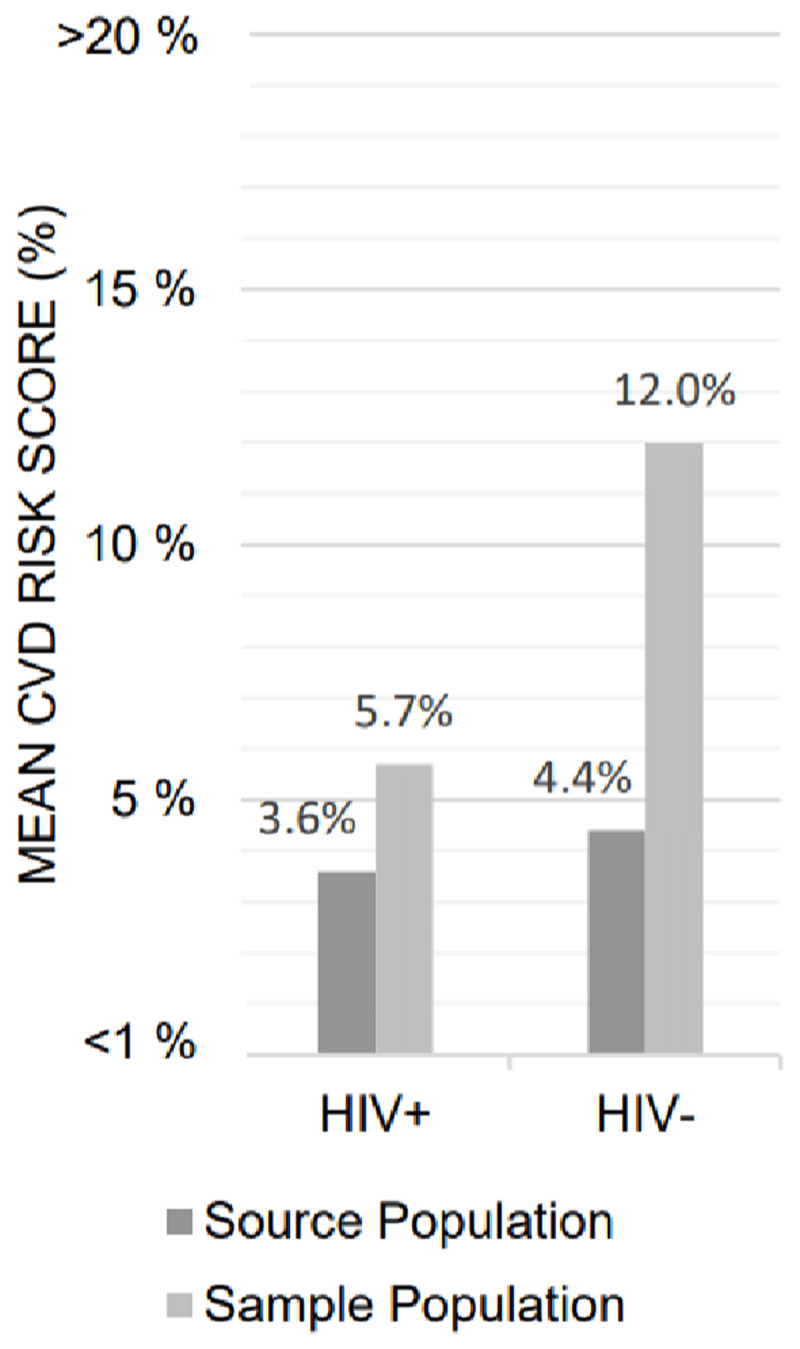
Mean CVD Risk Score Differences Between HIV+ and HIV- Source and Sample 35–49 Populations using the Framingham CVD Risk Score Scale.

Figure 2 illustrates the mean CVD risk score difference between participants sampled in each of the 35–49 population sub-groups as compared to the means for the source population by HIV status. In a sub-sample of 10 participants from each of the 35–49 population sub-groups, with a mean CVD risk of 9.6% for participants living with HIV and 10.2% for HIV-negative participants, the prevalence of LVH identified by the Sokolow-Lyon criteria and the total QRS criteria were greater among participants living with HIV as compared to HIV-negative participants: 60% vs 40% using Sokolow-Lyon criteria and 90% vs 60% using QRS criteria, for participants living with HIV and HIV-negative participants respectively. The prevalence of arterial stiffness was greater among the participants living with HIV and at increasing cutoffs, shown in Table 3.

**Table 3:** ECG and Arterial Function Measurements in 35–49 Population Sub-sample.

	HIV + No. (%)	HIV- No. (%)

**Risk Score**	n = 10	n = 10
Risk Score (mean, SD in years)	9.6 (3.2)	10.2 (1.2)
**ECG Measurements**	n =1 0	n = 10
ECG interval values		
Heart rate (bpm, mean)	75.3	70.0
PR-interval (ms, mean)	150.4	152.2
QRS-interval (ms, mean)	85.4	92.6
QTc-interval (ms, mean)	427.0	428.0
ECG Abnormalities		
Signs of Previous MI	0 (0%)	0 (0%)
QTc Prolongation	0 (0%)	2 (20%)
LBBB	0 (0%)	0 (0%)
RBBB	0 (0%)	0 (0%)
First-degree AV block	0 (0%)	0 (0%)
Second-degree AV block	0 (0%)	0 (0%)
Third-degree AV block	0 (0%)	0 (0%)
LVH by diagnostic criteria		
Sokolow-Lyon	6 (60%)	4 (40%)
Total QRS	9 (90%)	6 (60%)
**Arterial Function Measurements** ^1^	n = 5	n = 7
Augmentation index (mean)	34.2	32.7
Augmentation index (>33%)	2 (40%)	3 (43%)
Pulse wave velocity (m/s, mean)	11	9
Arterial stiffness (pulse wave velocity) prevalence^2^		
≥9 m/s	4 (80%)	5 (71%)
≥10 m/s	4 (80%)	1 (15%)
≥11 m/s	2 (40%)	0 (0%)

ECG = Electrocardiogram; Bpm = beats per minute; ms = milliseconds; MI = Myocardial infarction; LBBB = Left bundle branch block (LBBB); RBBB = Right bundle branch block (RBBB); AV block: atrioventricular block; LVH = Left ventricular hypertrophy; m/s = meters per second. ^1^ Arterial function measurements admissible for use were captured in 74 participants (63%, out of 118 participants) in the original sample. In this sub-sample, five participants living with HIV and seven HIV-negative participants were selected. ^2^ Lower prevalence cut-offs include participants categorized at higher cut-offs.

## Discussion

Our pilot study demonstrates that the incorporation of ECG and arterial function measurements into routine research activities of a population-based cohort study was acceptable to cohort participants. LVH and arterial stiffness were found to be common conditions in both high-risk populations sampled in the study. In terms of HIV status, arterial stiffness at the 11 m/s cutoffs were found to be more common among participants living with HIV as compared to HIV-negative participants in the 35–49 population.

To our knowledge, our study is the first to focus on the acceptance of sub-clinical CVD measurements by cohort participants in a population-based cohort in sub-Saharan Africa. With a 2% difference to routine blood pressure measurements, our results show that both the ECG and arterial function were well accepted by cohort participants.

A high prevalence of LVH was found in both high-risk populations irrespective of HIV status. Direct comparisons of ECG abnormalities within populations sampled in sub-Saharan Africa from limited studies published are difficult due to the wide variety in sampling and data collection methods used, particularly in the interpretation and presentation of ECG abnormalities. Broadly, however, our finding is consistent with published research that LVH is common in sub-Saharan Africa [28,29,30,31,32]. Uncontrolled hypertension, a known important risk factor for LVH, is a possible explanation for the high prevalence of LVH [33,34]. In our study, prevalence rates of hypertension ranged from 27% to 64% in both high-risk populations, and it is well-known from previous studies that both knowledge of hypertension status as well as control of hypertension is inadequate within sub-Saharan Africa [35].

In the secondary analysis focused on understanding the potential impact of mean CVD risk score in a small sub-sample, higher rates of LVH were found in participants living with HIV as compared to HIV-negative participants in the 35–49-year population. Overall, studies comparing ECG abnormalities by HIV status in sub-Saharan Africa are limited with conflicting findings. Two studies conducted in Nigeria have found higher rates of ECG abnormalities among people living with HIV (on treatment and treatment-naïve) as compared to the general population [36,37]. A study conducted in Uganda, however, found no statistical difference in the prevalence of ECGs with signs of ischemia or other abnormalities among people living with HIV on treatment as compared to the general population [38]. Outside of sub-Saharan Africa, a large study from North America on 4,518 participants living with HIV found a two to three fold increase of ECG abnormalities among people living with HIV as compared to estimates on the general population derived from other reported studies using the same ECG classification methods [39]. To what extent, if any, and how HIV infection or treatment could cause ECG abnormalities has not been established and should be investigated in another study.

Arterial stiffness, specifically at higher cut-off values, was more common among participants living with HIV as compared HIV-negative participants in the 35–49 population in our study. This finding is supported by both the primary and secondary analyses. A difference was not found in the 50+ population and might be due to the low capture of participants in this population (29 of 51 possible participants). A higher prevalence of arterial stiffness among people living with HIV is broadly consistent with other studies that have used other measurement tools (Sphygmocor Vx system) or metrics (ankle brachial index) to estimate arterial stiffness [40,41,42]. Direct effects of HIV replication on the endothelium, HIV-accelerated inflammatory processes and/or immunodeficiency are possible HIV specific mechanisms that increase atherosclerosis and increase arterial stiffness. However, given that no statistical difference was found among the 50+ population, further research aimed at understanding arterial stiffness in this age population is needed.

Our pilot study has several potential limitations. The sampling method used to select participants by HIV status among the 35–49 population resulted in a difference in the mean CVD risk score, with participants living with HIV having a lower CVD risk score as compared to the HIV-negative participants. To mitigate this fact, we conducted a sub-analysis with comparable mean CVD risk scores on the sample collected to understand the potential impact of the difference on ECG abnormalities and arterial stiffness outcomes. Finally, information regarding HIV treatment parameters, specifically type of ART and duration of treatment, were not collected, and could have informed findings related to participants living with HIV.

## Conclusions

The capture of sub-clinical markers of ECG abnormalities and arterial stiffness is an area in which large HIV focused population-based cohorts can play a critical role in providing information on the CVD burden across many sub-Saharan African settings. In addition to further expanding upon the acceptance and feasibility of ECG and arterial stiffness measurements by research staff, research exploring different CVD risk-stratified recruitment strategies for the implementation of sub-clinical measurements would be of value. For example, diabetes has been in focus with several ECG abnormality prevalence studies conducted in the region [28,29,30]. Diabetes and hypertension are of interest as both diseases have been identified as common CVD risk factors in sub-Saharan Africa and can relatively easily be screened in cohort field activities [43,44,45].

LVH and arterial stiffness are both common irrespective of HIV status, with arterial stiffness potentially more common among people living with HIV. These findings have implications for the design of studies that explore further to understand the feasibility and costs of incorporating sub-clinical markers into cohorts, confirm and explore the reasons for high rates of LVH and arterial stiffness and finally describe the extent, if any, HIV infection or treatment can cause ECG abnormalities and arterial stiffness.

## Data Accessibility Statement

The data that support the findings of this study are available from the corresponding author, upon reasonable request.
